# Aerobic fitness and cognitive function in older African Americans: The RAATE studies

**DOI:** 10.1002/alz70860_102868

**Published:** 2025-12-23

**Authors:** Owen T. Carmichael, Angelique Litsey, Neil Johannsen, Robbie Beyl, Robert L Newton

**Affiliations:** ^1^ Pennington Biomedical Research Center, Baton Rouge, LA, USA; ^2^ Louisiana State University, Baton Rouge, LA, USA

## Abstract

**Background:**

Regular engagement in physical activity, and engagement in interventions that increase aerobic fitness, are associated with better cognitive performance among older adults. But the status of aerobic fitness as a risk factor for poor cognitive function is unclear, and studies in this area have not featured large numbers of older African Americans.

**Method:**

The Reducing African Americans’ Alzheimer's Disease Risk Through Exercise (RAATE) Study randomized 129 initially sedentary, cognitively healthy, older African American adults to one year of aerobic exercise training or successful aging education. At study entry, all participants completed a graded sub‐maximal treadmill aerobic exercise test to at least 80% age‐predicted heart rate maximum, as well as the NIH Toolbox cognitive battery and the Rey Auditory Verbal Learning Test (RAVLT). Baseline aerobic fitness, quantified by exercise‐test‐based peak VO2 (ml/kg/min), was related to baseline cognitive scores in linear regression models controlling for age and sex.

**Results:**

A total of 119 individuals (mean age 68 +/‐ 5 years; 76% female; 34% with income < $50k/year; 45% married) provided requisite baseline assessment data. Greater peak VO2 was associated with greater NIH Toolbox global composite score (β=.38 score units per unit ml/kg/min; *p* = .045) and greater dimensional change card sort score (β=.69 score units per unit ml/kg/min, *p* = .035). In stratified analyses, greater peak VO2 was associated with greater pattern comparison processing score (β=.97 score units per unit ml/kg/min, *p* = .012) in men only. Other associations between peak VO2 and cognitive scores were not significant.

**Conclusion:**

Greater aerobic fitness was associated with greater global cognitive function in cognitively‐healthy older African American exercise trial participants. Possible sex‐ and domain‐specific associations between aerobic fitness and cognitive function should be further explored in this population.

